# Global trends and hotspots in pain associated with bipolar disorder in the last 20 years: a bibliometric analysis

**DOI:** 10.3389/fneur.2024.1393022

**Published:** 2024-05-23

**Authors:** Hong Qing Zhao, Mi Zhou, Jia Qi Jiang, Zhi Qiang Luo, Yu Hong Wang

**Affiliations:** ^1^Science and Technology Innovation Center, Hunan University of Chinese Medicine, Changsha, China; ^2^School of Pharmacy, Hunan University of Chinese Medicine, Changsha, China; ^3^Department of Graduate School, Hunan University of Chinese Medicine, Changsha, China

**Keywords:** bipolar disorder, pain, bibliometric analysis, CiteSpace, VOSviewer, R package

## Abstract

**Purpose:**

The prevalence of comorbid pain and Bipolar Disorder in clinical practice continues to be high, with an increasing number of related publications. However, no study has used bibliometric methods to analyze the research progress and knowledge structure in this field. Our research is dedicated to systematically exploring the global trends and focal points in scientific research on pain comorbidity with bipolar disorder from 2003 to 2023, with the goal of contributing to the field.

**Methods:**

Relevant publications in this field were retrieved from the Web of Science core collection database (WOSSCC). And we used VOSviewer, CiteSpace, and the R package “Bibliometrix” for bibliometric analysis.

**Results:**

A total of 485 publications (including 360 articles and 125 reviews) from 66 countries, 1019 institutions, were included in this study. Univ Toront and Kings Coll London are the leading research institutions in this field. J Affect Disorders contributed the largest number of articles, and is the most co-cited journal. Of the 2,537 scholars who participated in the study, Stubbs B, Vancampfort D, and Abdin E had the largest number of articles. Stubbs B is the most co-cited author. “chronic pain,” “neuropathic pain,” “psychological pain” are the keywords in the research.

**Conclusion:**

This is the first bibliometric analysis of pain-related bipolar disorder. There is growing interest in the area of pain and comorbid bipolar disorder. Focusing on different types of pain in bipolar disorder and emphasizing pain management in bipolar disorder are research hotspots and future trends. The study of pain related bipolar disorder still has significant potential for development, and we look forward to more high-quality research in the future.

## 1 Introduction

Bipolar Disorder (BD) is a severe mental health condition characterized by significant emotional fluctuations, including alternating periods of mania or hypomania and depression (1). Clinically, BD is often categorized into several subtypes, with the most common being Bipolar Disorder Type I and Type II. Bipolar Disorder Type I is typically characterized by full-blown manic episodes and periods of depression, whereas Type II is marked by hypomanic and depressive episodes. Additionally, there are other subtypes such as cyclothymic disorder (2).

The global lifetime prevalence of bipolar disorder is approximately 1–2.5%, with certain variations across different regions and populations. The disorder typically begins in late adolescence or early adulthood, but diagnosis is often delayed due to its early symptoms being confused with major depressive disorder (3, 4).

Bipolar Disorder is linked to factors like neurotransmitter imbalances in the brain, neuroinflammation, oxidative stress, and anomalies in neurodevelopment (5). The cyclical nature and emotional fluctuations of the disorder pose significant harm to patients, who may exhibit high-risk behaviors during manic phases and experience profound sadness and despair during depressive episodes. Approximately 25–50% of patients may attempt suicide, and about 15% may die by suicide (6, 7). Moreover, Bipolar Disorder is also associated with a higher comorbidity rate (8), including cardiovascular diseases (9), endocrine disorders (10), and metabolic syndrome (11).

Pain, as a frequent comorbidity among bipolar disorder patients, has garnered growing interest in recent years. Pain is often considered a subjective discomfort, but increasing evidence shows a deeper interaction between pain and bipolar disorder (12). Research indicates that patients with bipolar disorder often suffer from chronic pain, including neuropathic and musculoskeletal pain (13). This pain frequently coincides with the mood swings of bipolar disorder, particularly during depressive episodes, when the intensity and frequency of pain seem more pronounced (14). This suggests that the emotional fluctuations of bipolar disorder may increase an individual’s sensitivity to pain.

Pain represents more than just biological phenomena, it may also trigger BD. Research indicates that chronic pain can alter neurotransmitter levels (15), impacting emotional regulation and intensifying Bipolar Disorder symptoms. Furthermore, chronic pain is linked to changes in the activity of brain areas responsible for stress response and emotional regulation (16).

Neglecting pain in the treatment of bipolar disorder can lessen the treatment’s efficacy. Comprehending and managing pain in BD plays a crucial role in enhancing treatment compliance, reducing recurrence, and improving patient quality of life. Concentrating on pain research in patients with bipolar disorder is instrumental in uncovering its pathophysiological foundations and in advancing more effective treatment strategies.

Bibliometrics is a quantitative research method aimed at systematically analyzing the distribution, growth, and developmental trends of academic literature. Utilizing bibliometrics, scholars can gain a clearer perception of a field’s current research landscape, prominent research areas, and prospective research directions.

Regrettably, there are currently no bibliometric studies that have explored the roles of pain in bipolar disorder. Given the context, this article employs bibliometric analysis to holistically assess existing research on pain-related bipolar disorder, aiming to identify research trends, key findings, existing gaps, and potential future directions.

## 2 Materials and methods

### 2.1 Search strategy

**Figure 1:**
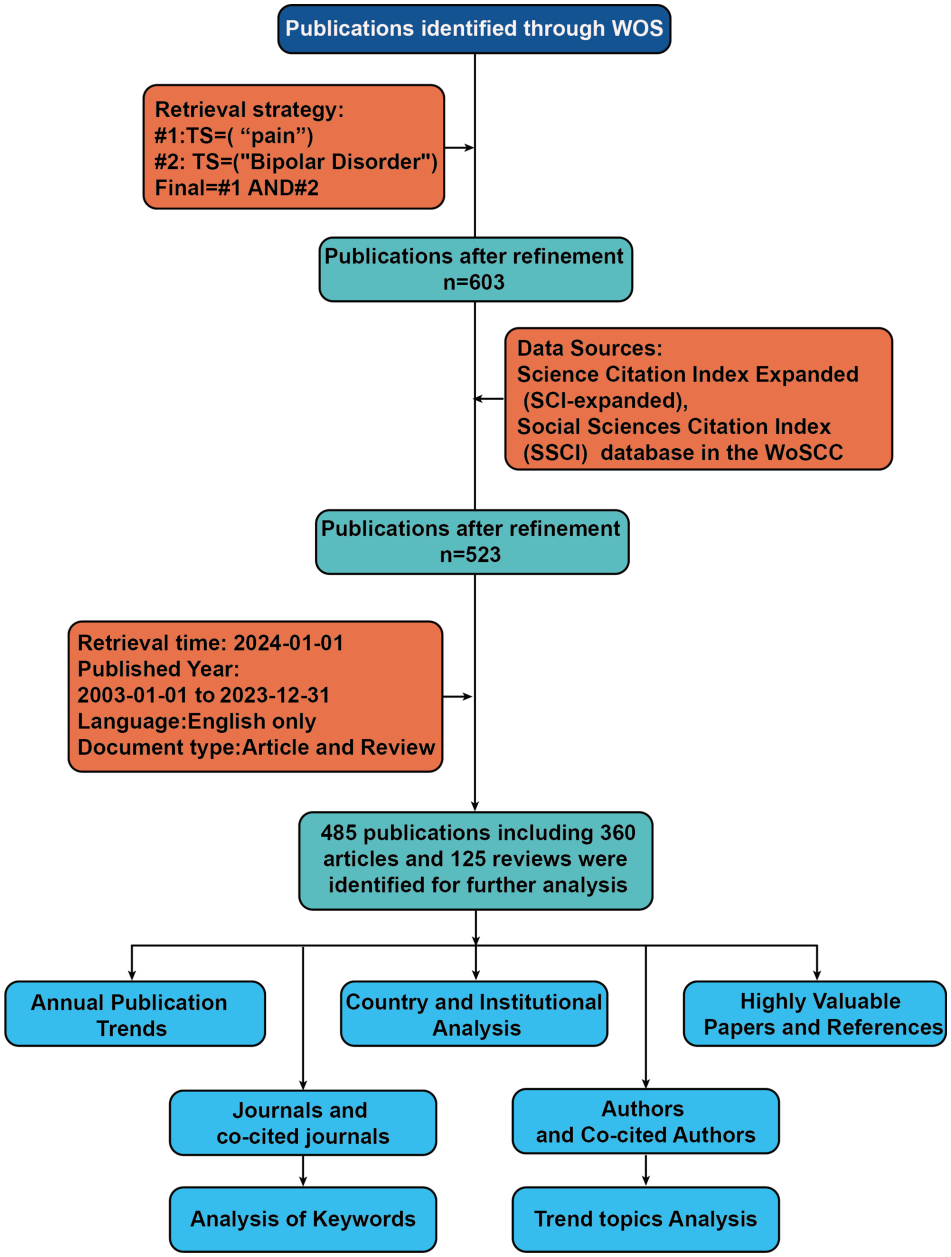
Flowchart of literature identification and analysis process. TS, Topic; WOS, web of science.

We selected the Web of Science Core Collection (WoSCC) database to conduct a literature search on 2024-01-01. We referenced existing bibliometric articles on pain research and those on bipolar disorder to establish the following search strategy (17–20): #1:TS = (“pain”), #2:TS = (“Bipolar Disorder”), final = #1 AND #2. LA = (English), and the type of documents was set to “articles” and “review.” The publication period was specified as 2003-01-01 to 2023-12-31. Following the initial retrieval, we screened the titles and abstracts to confirm the eligibility of the articles based on predefined inclusion and exclusion criteria. Finally, a total of 485 references were obtained. We saved the document data in the form of full records and cited references as plain text format. The flowchart of the screening process is shown in Figure 1.

### 2.2 Data analysis

Leveraging the capabilities of VOSviewer (version 1.6.18), a bibliometric analysis software of substantial renown (21), we facilitated the generation of visualizations representing cooperative, co-citation, and co-occurrence networks. A co-citation connection occurs when a scholarly article simultaneously references two or more distinct works, be they papers, journals, or authors. This linkage is perceived as foundational in forming a network of specific research domains, facilitating an analysis of the depth and nature of the interrelationships among these specialized fields (22–24). The analyses conducted in this study utilizing VOSviewer encompassed cooperative network of nations, institutions and authors; co-occurrence analysis of keywords; as well as co-citation analysis of journals and authors.

**Figure 2:**
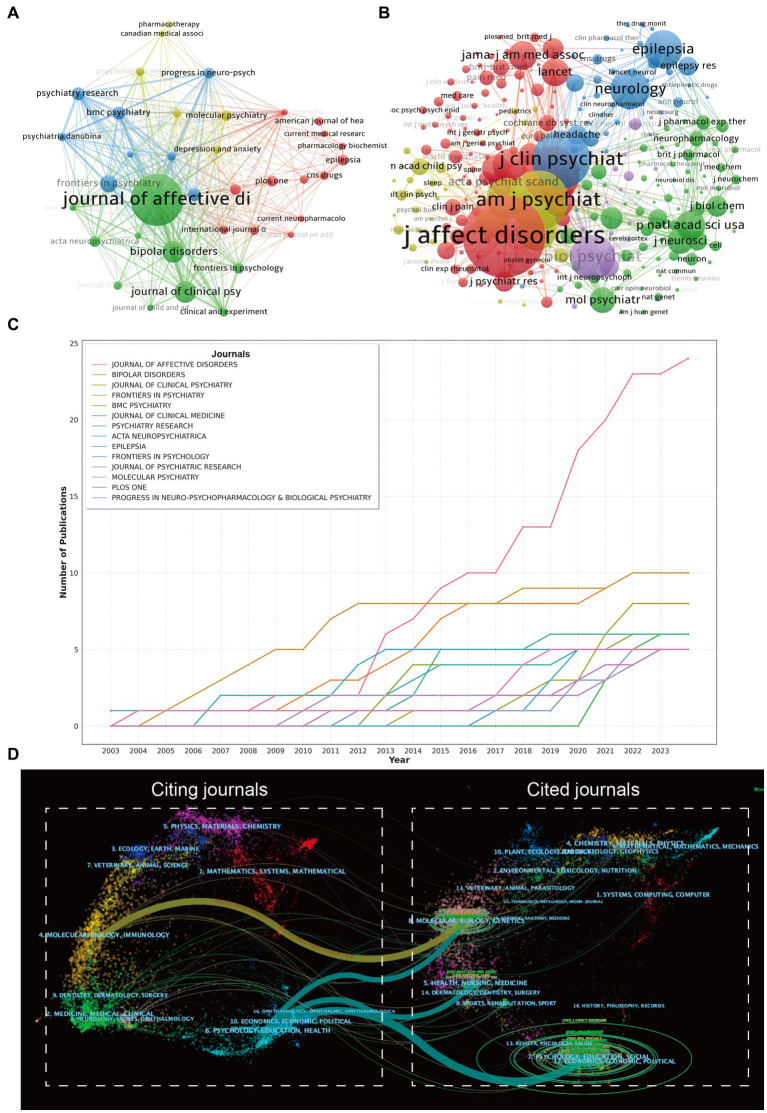
**(A)** The visualization of journals cooperation networks based on Citespace. **(B)** and network visualization of co-cited journals based on VOSviewer. **(C)** Top 10 journals’ production over time. **(D)** The dual-map overlay of journals related to pain-related Bipolar Disorder, the overlay segments into two main areas: journals citing others on the left, and journals being cited on the right, connected by a trajectory curve representing citation paths. Ellipses in the diagram denote the publication volume of each journal, with the ellipse’s width indicating the diversity of contributing authors and its height reflecting the total number of articles published by the journal.

We also engaged CiteSpace (version 6.1. R1), an alternative software for bibliometric analysis and visualization (25), devised by Professor Chen Meichao of Drexel University. Serving as a valuable metric, burst citations highlight references (or keywords) that have captured academic interest within a specified field during a certain timeframe (26). With the assistance of CiteSpace, we pinpointed references and keywords exhibiting high citation bursts. The dual-map overlay of journals delineates the positioning of research within the broader spectrum of principal research disciplines, captures the informational flow at the journal level, and offers a visual representation of the discipline’s research dynamics (27, 28). Utilizing CiteSpace, we crafted a dual map overlay of journals pertaining to the pain-related bipolar disorders, as illustrated in Figure 2D.

We used the R package “Bibliometrix” (version 3.2.1)^1^ to illustrate the annual publication volume trends in this research field and the publication output of various countries, which not only showcases the academic output of these countries but also elucidates the state of international collaboration among them. Subsequently, we created trend graphs of cumulative publication volumes for the top 10 institutions and journals, which further revealed the influence of major institutions and journals within the field. Moreover, we conducted a detailed analysis of trend topics using the “Bibliometrix” package.

### 2.3 Procedures for analysis

We applied STATA to perform a regression model for the growth of world publication in pain-related bipolar disorder.

Full records and cited references of the retrieved articles were downloaded from the WoSCC database and saved as .txt format for below analysis.

#### 2.3.1 R package “bibliometrix”

**Figure 3:**
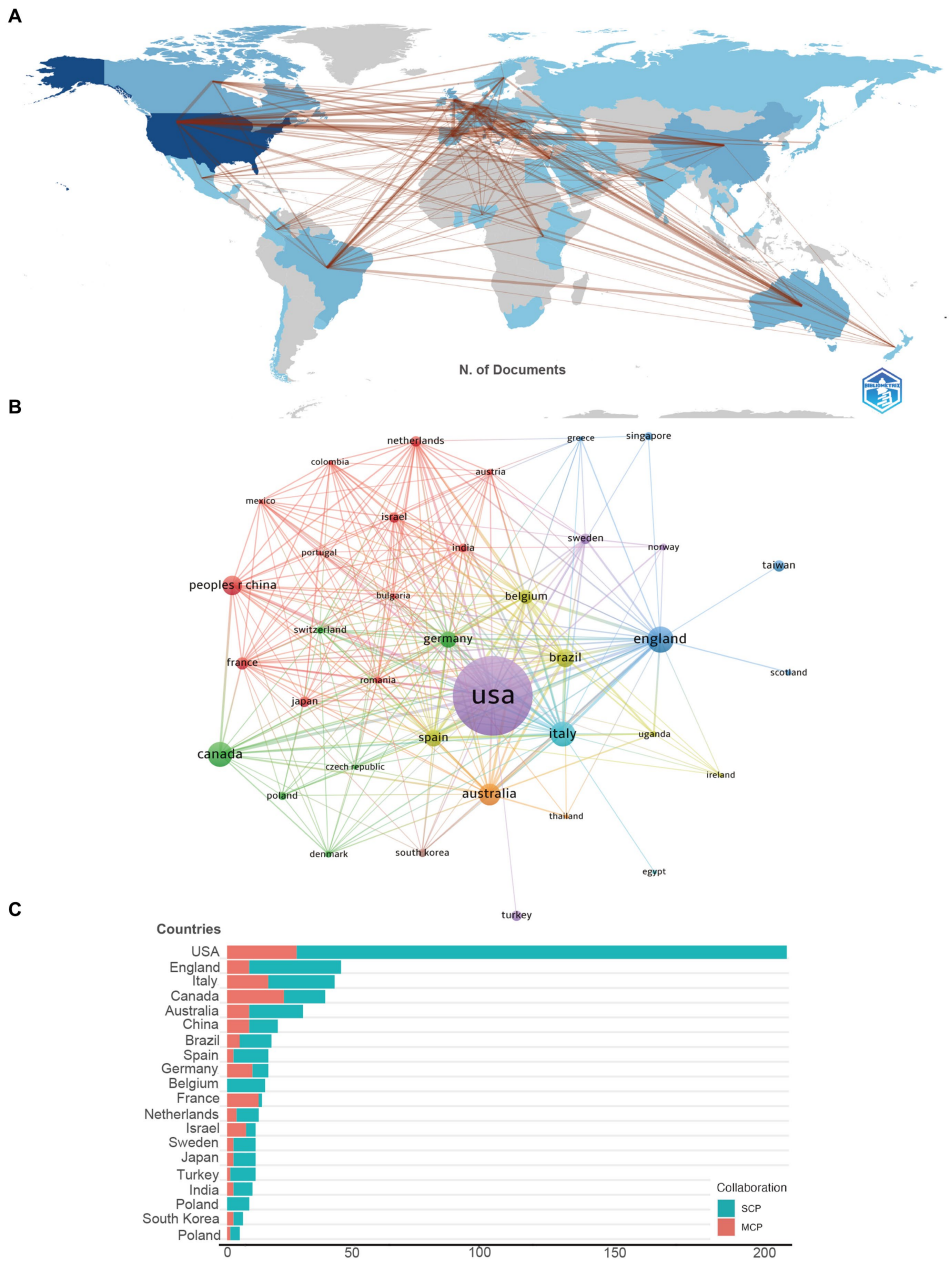
**(A)** The geographical network map of pain-related bipolar disorder. **(B)** The overlay visualization map of country co-authorship analysis conducted by VOSviewer. **(C)** TOP 20 Corresponding Author’s countries that produced the largest number of literature (SCP, Single Country Publications; MCP, Multiple Country Publications).

**Figure 4:**
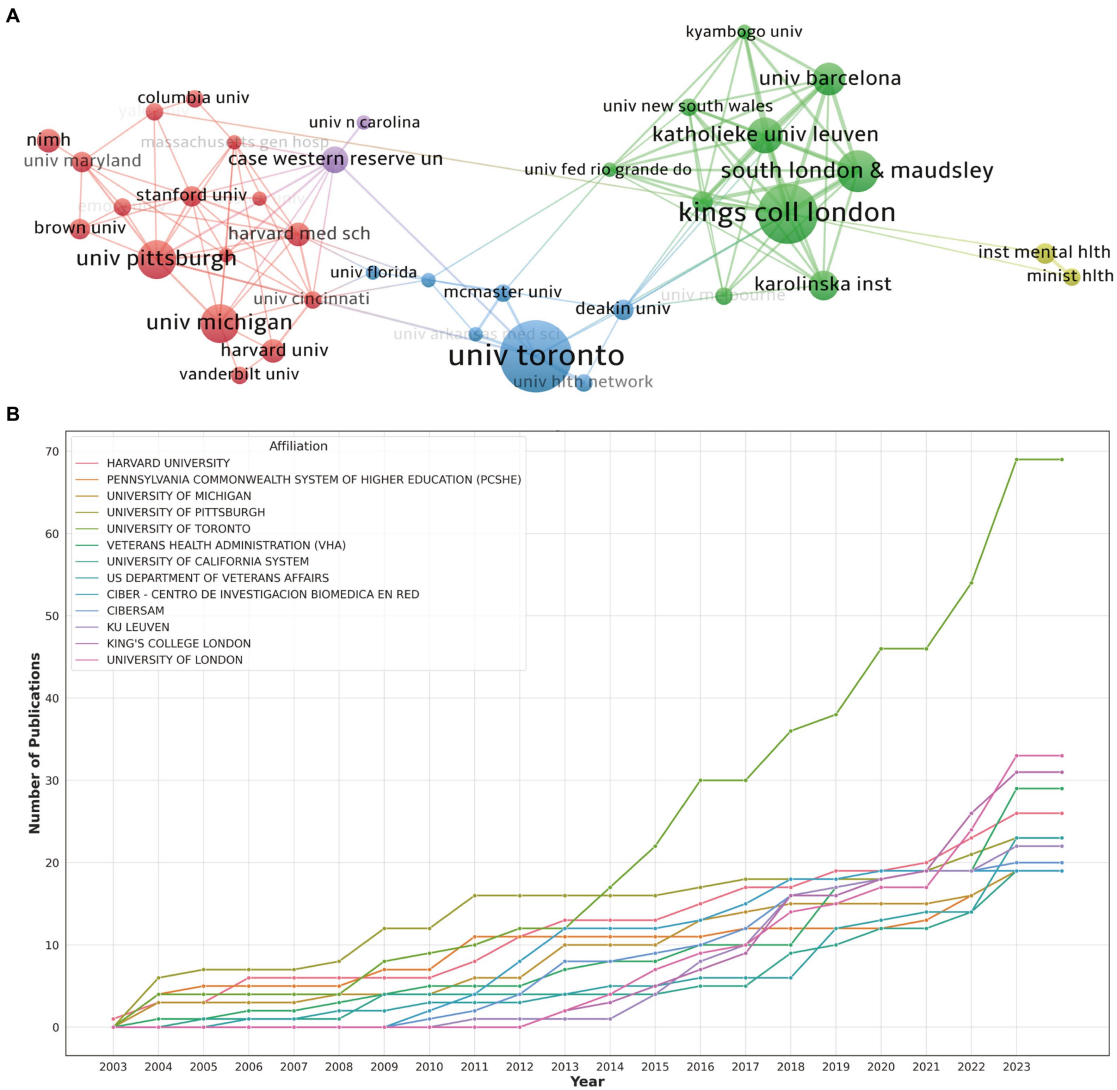
**(A)** The visualization of institutions cooperation networks based on VOSviewer. **(B)** Top 10 institutions’ production over time.

**Figure 5:**
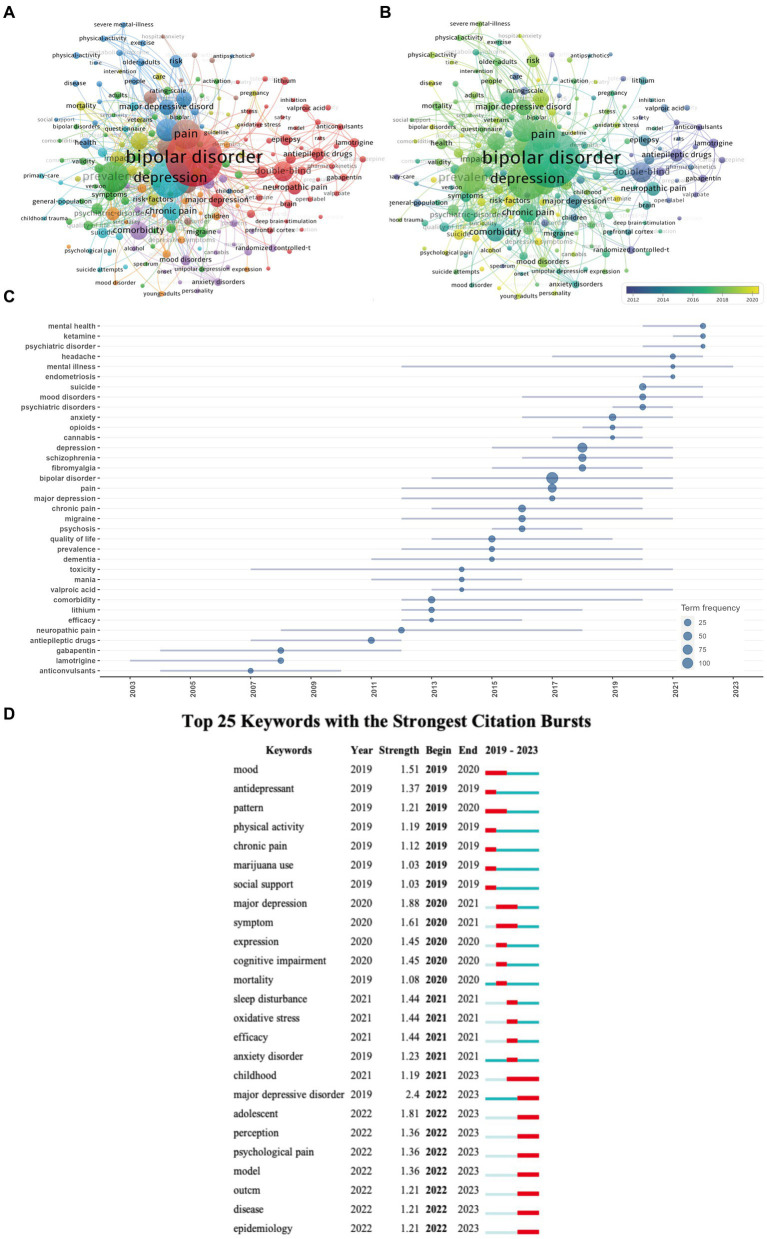
**(A)** Visualization map of keywords co-occurrence network. **(B)** Overlay visualization of the keywords network based on VOSviewer. and Trend Topics Analysis **(C)** of pain-related bipolar disorder research. **(D)** Top 25 keywords with the strongest citation bursts based on Citespace.

In utilizing the “bibliometrix” package in R Studio for bibliometric analysis, the process begins with executing the biblioshiny () function to upload data via a web interface. For collaborative mapping among countries, set the parameters to a minimum of three connections and an edge size of 2.1 (Figure 3A). In the visualization of the Corresponding Author’s countries, set the number of countries to 20 (Figure 3C). The analysis then focuses on the top 13 institutions by publication volume, outlined in Figure 4B, and extends to the top 14 journals, depicted in Figure 2C. Lastly, to discern trending topics, adjust settings for a word frequency threshold of five and select three significant words per year, enabling an insightful delineation of research trends (Figure 5C).

Detailed information about how to create maps by using CiteSpace and VOSViewer are in the manuals of the software (21, 25).

#### 2.3.2 VOSviewer

**Figure 6:**
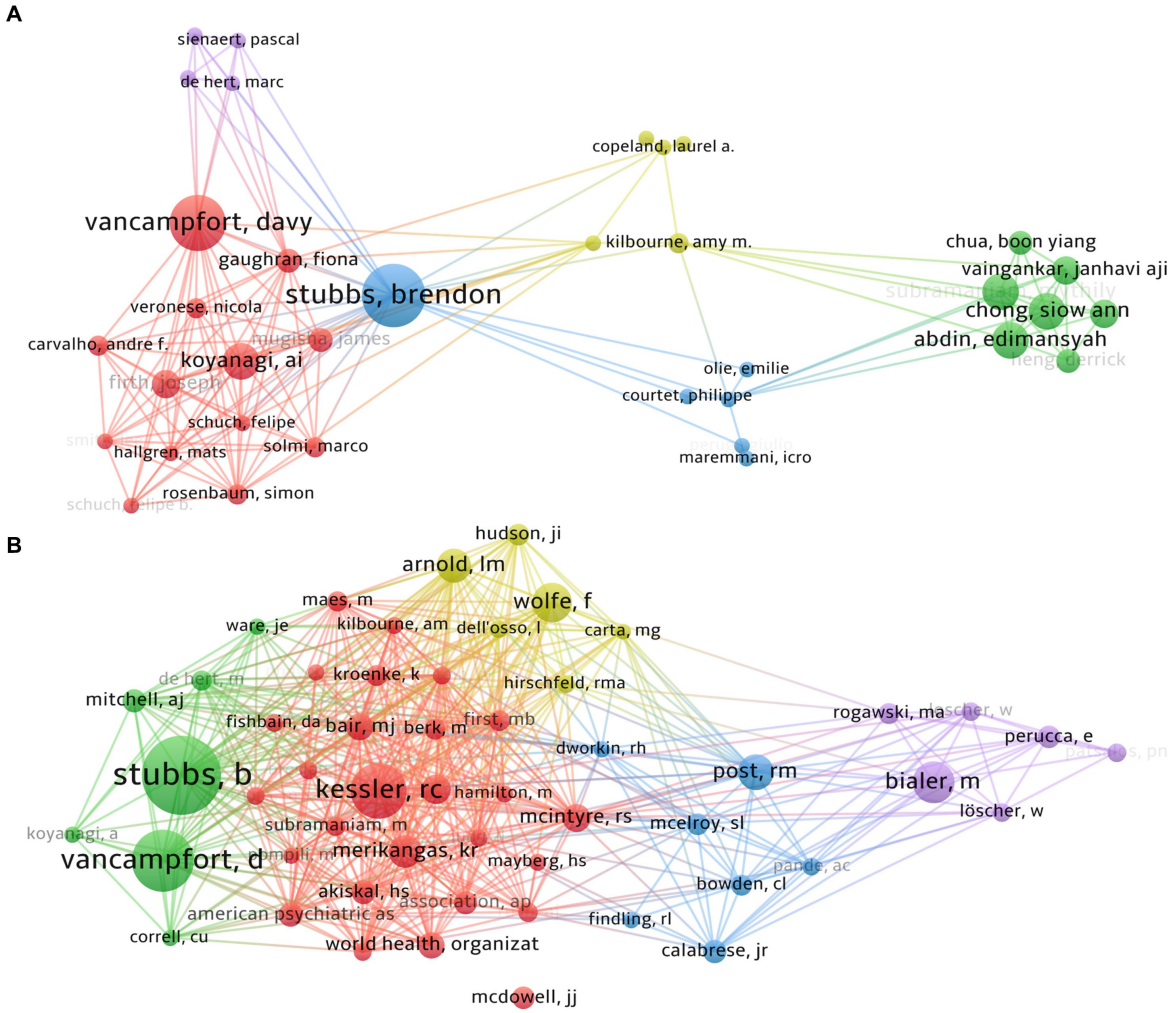
The visualization of authors **(A)** and co-cited authors **(B)** cooperation networks based on VOSviewer.

In the visualization of country cooperation relationships, we set a threshold of a minimum of 5 publications, resulting in 28 countries (out of 66) meeting the criteria (Figure 3B). In the analysis of institutional cooperation networks, out of 1019 institutions, 45 had a publication count of at least 5 (Figure 4A). For the journal cooperation network, setting a threshold of at least 3 publications identified 37 journals (out of a total of 298) that qualified (Figure 2A). The co-cited journal network visualization used a minimum of 20 citations as a threshold, with 276 journals (out of 5,325) meeting the standard (Figure 2B). In the visualization of the author and co-cited author collaboration networks, we set thresholds of a minimum of 4 publications per author and 20 citations per author, respectively. The findings show that among 2537 authors, only 20 satisfied the publication threshold (Figure 6A), while among 19,989 co-cited authors, 53 met the citation threshold (Figure 6B). For keyword co-occurrence analysis, a threshold of at least 5 co-occurrences was set, with 193 keywords (from a total of 2,869) meeting the standard (Figure 5A), and an overlay visualization of keywords was conducted, see Figure 5C.

#### 2.3.3 CiteSpace

CiteSpace utilizes a variety of structural and temporal metrics to analyze document or author co-citation references and the clusters they form. This research focuses on two specific metrics: Burst and Sigma, as identified by Chen and Song (28). The burst detection algorithm is a computational method designed to identify significant surges in the citation count of specific references, signaling abrupt shifts in event patterns. Meanwhile, the Sigma (Σ) value serves as an indicator of scientific innovation (28).

During our analysis with CiteSpace software, we applied the following selection criteria: G-index set to 25; Link Retaining Factor (LRF) at 3.0; Look Back Year (LBY) of 5 years; and the percentage of marked nodes at 1.0%.

**Figure 7:**
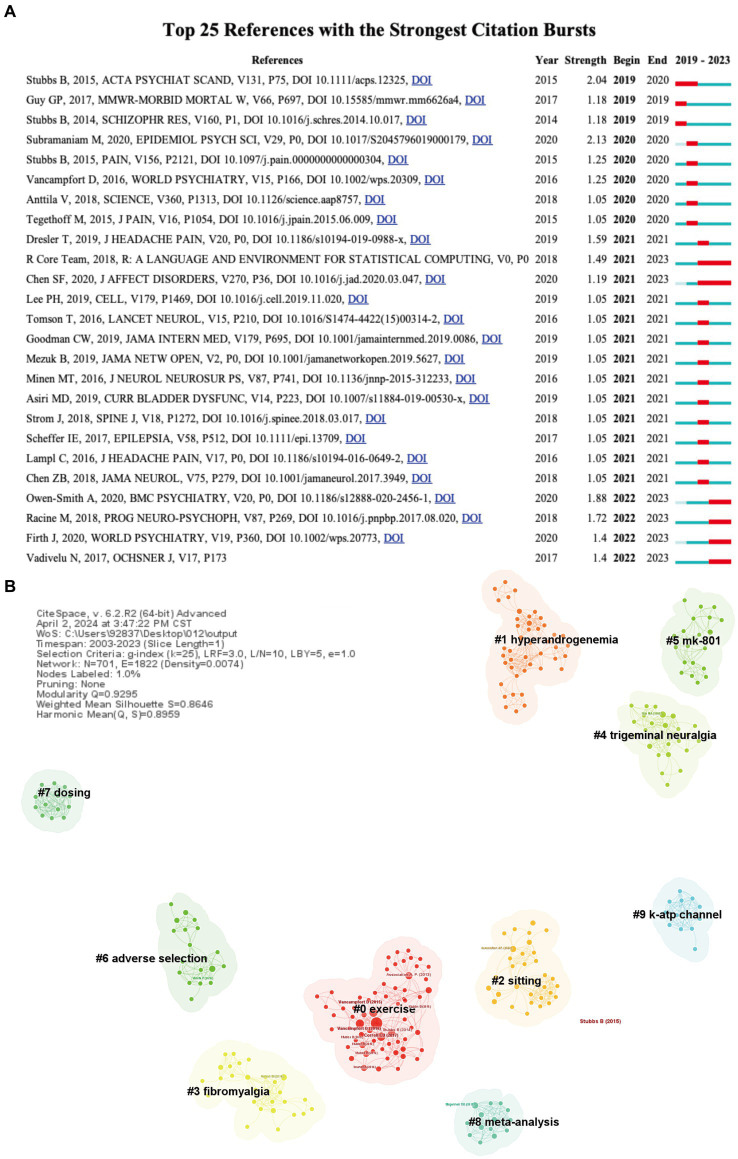
**(A)** Top 25 references with strongest citation bursts of publications and **(B)** the landscape mapping view of cluster based document co-citation analysis (DCA), generated by g-index (*k* = 25) per slice between 2003 and 2023 (LRF = 3.0, L/N = 10, LBY = 5, *e* = 1.0). Each color represents different cluster..

For the burst analysis of references (Figure 7A) and the strong burst analysis of keywords (Figure 5D), we configured a specific detection model: f(x) = αe^-αx^, α_1_/α_0_ = 0.2, α_i_/_αi-1_ = 0/2; The Number of States = 2; ү = 0.2; Minimum Duration = 2.

Regarding the specific parameters for the DCA analysis, they are thoroughly described in Figure 7B, further details will not be provided here.

## 3 Results

### 3.1 Annual publication trends

**Figure 8:**
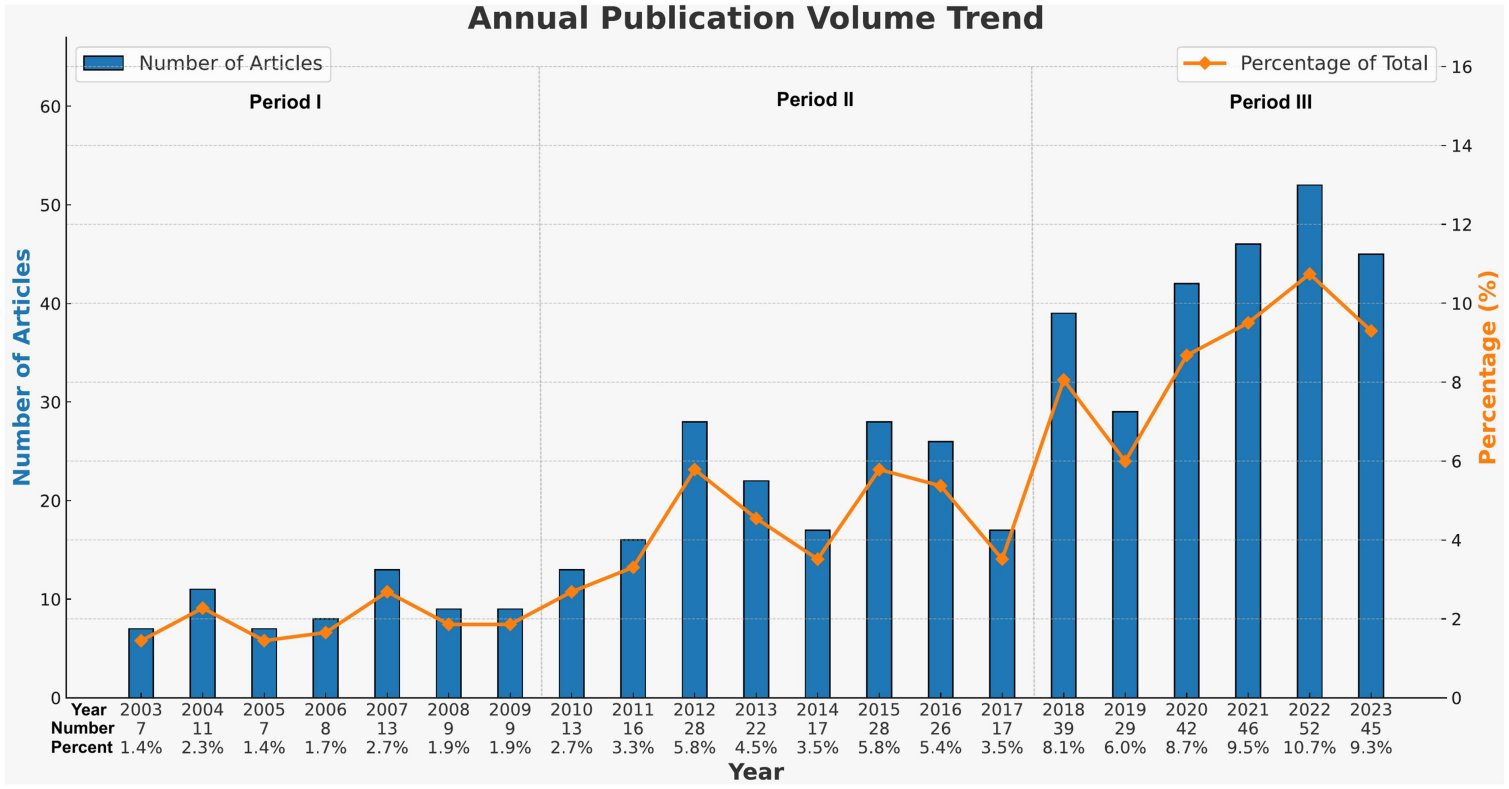
Annual outputs of publications regarding pain-related bipolar disorder.

Considering the yearly increase in publication numbers, the entire period can be divided into three phases: Phase I (2003–2009), Phase II (2010–2017), and Phase III (2018–2023). As shown in Figure 8, the number of publications in Phase I was relatively low, with an average annual publication count of about 9.1, representing the initial stage of research on pain-related Bipolar Disorder. Entering Phase II, the number of publications began to gradually increase, with an average annual publication count of approximately 20.8. In the past 6 years (Phase III), the number of publications began to increase significantly, with an average annual publication count of about 42.2, marking a substantial rise compared to the earlier two phases. In 2018, the number of related publications was 39, 2.3 times that of 2017. This trend demonstrates the growing recognition among scholars of the significance of pain-related bipolar disorder.

### 3.2 Country and institutional analysis

A total of 1019 institutions, and 66 nations have contributed to this collective body of literature. The top 10 contributors hail from diverse regions encompassing Asia, Europe, and North America (Figure 3A). Table 1 discloses that a predominant portion of publications originates from USA (226) and England (47), collectively accounting for a commanding 56.29% of the total global publications. Hot on their heels are Italy (*N* = 44, 9.07%), Canada (*N* = 43, 8.86%), and Australia (*N* = 36, 7.42%).

**Table 1:** Top 10 countries and institutions on research of pain-related Bipolar Disorder.

Rank	Country	Articles	Citation	Institution	Counts
1	USA	226	10,190	Univ Toronto	24
2	England	47	1,623	Kings Coll London	20
3	Italy	44	1,654	South London and Maudsley Nhs Fdn Trust	14
4	Canada	43	1,772	Univ Pittsburgh	13
5	Australia	36	970	Univ Michigan	13
6	China	31	928	Univ Pisa	13
7	Brazil	28	992	Katholieke Univ Leuven	12
8	Spain	25	1,373	Univ Barcelona	11
9	Germany	24	1,205	Karolinska Inst	10
10	Belgium	19	794	Case Western Reserve Univ	9

Further, Table 1 accentuates that the publications from USA boast the highest cumulative citation frequency (10190), followed distantly by those from Canada (1,772), Italy (1,654), and England (1,623). In Figure 3B, lines are utilized to represent the frequency of international academic collaboration, with the node’s size signifying each country’s publication tally. The visual representation in Figure 3B underscores a thriving international research collaboration landscape, with Canada, the United States, and Italy engaging in dynamic cooperative efforts. A close collaborative synergy between England and Italy is also discernible.

Although the USA leads in publication volume by a significant margin compared to other countries, the proportion of its multi-country publications (MCP) is relatively low in comparison to its domestic research output (Figure 3C). This suggests that in this field of research, there is a notable lack of academic collaboration between the USA and other countries.

Figure 4A offers a graphical delineation of the inter-institutional collaboration network, accentuating the strong ties that exist among a diverse array of institutions. As suggested by Figure 4A’s visualization, considerable collaboration is apparent between South London and & Maudsley and Kings Coll London, as well as between Univ Pittsburgh and Univ Michigan.

Figure 4B, alongside Table 1, ranks the top 10 institutions by their contributions to the literature. Univ Toront leads the cadre with 24 publications, closely followed by Kings Coll London and South London and & Maudsley Nhs Fdn Trus with 20 and 14 publications, respectively. Notably, the disparity in publication volume across these institutions does not emerge as significant.

However, it’s pivotal to note that these collaborations principally take place within each institution’s home country, revealing a striking paucity of vibrant academic collaborations between institutions from disparate nations.

Figure 3B showcases a promising incline in the annual publication output from the top 10 institutions in recent years. Univ Pittsburgh blazed the trail by being the first to contribute to this field, but its cumulative document count appears to have plateaued starting from 2011. Conversely, although Univ Toronto joined the field later, it has witnessed a swift escalation in publication output beginning from 2014.

### 3.3 Journals and co-cited journals

Harnessing the capabilities of VOSviewer, we curated a visual representation of journals and co-cited journals within this research realm. The visualization of journals cooperation networks was shown in Figure 2A, the size of the nodes represents the publication volume of each journal.

When a journal concurrently cites two or more other journals, it establishes a co-citation relationship between them (22). The dimension of a node corresponds to the cumulative citation count of the journal, and a linkage between any two nodes illustrates the co-citation association they share. Journals depicted by enlarged nodes signify a substantial citation tally, denoting their pivotal influence within an academic discipline. Figure 2B unfurls a network map of co-cited journals, featuring those commanding a minimum of 20 citations. As explicated in Figure 2B, 276 co-cited journals were displayed, reflecting the aggregate link strength. Total link strength (TLS), the sum of co-citation frequencies between two journals, indicating the strength of their connection within the literature. The five most frequently co-cited journals, exhibiting the most formidable total link strength (TLS), comprised: J affect disorder (TLS = 41,311), J clin psychiat (TLS = 31,529), Am J Psychiat (TLS = 31,377), Pain (TLS = 29,854), and Biol Psychiat (TLS = 28,541) (refer to Table 2).

**Table 2:** Top 10 journals and co-cited journals for pain-related Bipolar Disorder.

Ranks	Journals	Count	IF	Q	Co-cited Journals	TLS	IF	Q
1	J Affect Disorders	24	6.6	Q1	J Affect Disorders	41,311	6.6	Q1
2	Bipolar Disorders	10	5.4	Q1	Am J Psychiat	31,377	17.7	Q1
3	J Clin Psychiat	10	5.3	Q1	J Clin Psychiat	31,524	5.3	Q1
4	Frontiers In Psychiatry	8	5.4	Q2	Pain	29,854	7.9	Q1
5	BMC Psychiatry	6	4.4	Q2	Biol Psychiat	28,541	10.6	Q1
6	J Clin Medicine	6	3.9	Q2	Arch Gen Psychiat	20,618	N	N
7	Psychiatry Research	5	11.3	Q1	Bipolar Disorders	21,376	5.4	Q1
8	Acta Neruopsychiatrica	5	3.8	Q2	Neurology	23,870	9.9	Q1
9	Frontiers In Psychology	5	4.2	Q2	Epilepsia	21,284	5.6	Q1
10	Epilepsia	5	5.6	Q1	Brit J Psychiat	13,564	10.5	Q1

IF, Impact Factor; Q, Journal Citation Reports (JCR) Quartile; TLS, Total Link Strength.

Figure 2C sketches the annual outputs of the top 14 journals spanning from 2003 to 2023. The publication volume in J Affect Disorders has experienced a steep ascent in recent years. Conversely, the publication growth in bipolar disorders has been relatively placid. Table 2 catalogs the top 10 most productive and co-cited journals incorporated in this inquiry. J Affect Disorders (impact factor = 6.6, 2023) surfaced as the preeminent publisher, boasting 24 publications. Further, there were 10 publications in bipolar disorders (IF = 5.4, 2023), and J Clin Psychiat (IF = 5.3, 2023), 8 publications in Frontiers In Psychiatry (IF = 5.4, 2023). Five of the top 10 journals fell under the Q1 JCR region.

Local citations, deduced from the reference list, afford insight into their localized impact, whereas total citations mirror wider interest across various disciplines. Within this ranking, J Affect Disorders commandeered the list with 787 citations, followed by Am J Psychiat with 542 citations, and J Clin Psychiat with 524 citations (Figure 2B). This clearly indicates a high proportion of high-caliber publications within these journals.

A dual map overlay of journals pertaining to the pain-related bipolar disorders was illustrated in Figure 2D. Clusters residing on the left of the orange line designate citing journals, whereas the cluster to the right of the orange trajectory signifies co-cited journals.

There are four main citation paths. The yellow paths show that journals published in the fields of molecular/biology/immunology are usually influenced by journals published in the fields of molecular/biology/genetics. The blue paths indicate that journals published in the fields of psychology/education/health are typically influenced by journals published in the fields of molecular/biology/genetics, molecular/biology/genetics, health/nursing/medicine and psychology/education/social. The dual-map overlay of journals predicts that hot spots and trends of pain-related bipolar disorder will converge in the fields of psychology/education/health.

### 3.4 Authors and co-cited authors

In the exploration of pain-related bipolar disorder, 2537 researchers participated. The top 10 contributors collectively produced 85 publications, representing approximately 17.5% of the total output within this field (Table 3). Stubbs B emerged as the most productive author, with 16 publications, succeeded by Vancampfort D with 14 publications, and Abdin E with 8 publications (Table 3). The H-index, a metric designed to quantify a scholar’s impact who has authored H papers each garnering at least H citations, was employed to appraise the influence of the scientific investigations. As evidenced in Table 3, Stubbs B distinguished himself as the author boasting the highest H-index, followed by Vancampfort D.

**Table 3:** Top 10 authors and co-cited authors on research of pain-related Bipolar Disorder.

Rank	Authors	Counts	H-index	Co-cited authors	Citations	Total link strength
1	Stubbs B	16	12	Stubbs B	127	1098
2	Vancampfort D	14	10	Vancampfort D	96	830
3	Abdin E	8	6	Kessler RC	86	398
4	Chong Sa	8	6	Bialer M	62	352
5	Koyanagi A	8	7	Wolfe F	58	481
6	Subramaniam M	8	6	Post RM	51	691
7	Firth J	6	6	Arnold LM	49	447
8	Shafie S	6	5	Merikangas KR	46	266
9	Vaingankar JA	6	5	Beck AT	41	134
10	Bialer M	5	5	Mcintyre RS	39	301

VOSviewer provides a visualization of the interconnections among authors, as exhibited in Figure 6A. There exists a profound collaboration between Stubbs B and Vancampfort D as well as Koyanagi AI. Stubbs B and Vancampfort D co-authored two papers in this field, with the most cited article, titled “The prevalence of pain in bipolar disorder: a systematic review and large-scale meta-analysis,” being the first systematic evaluation and meta-analysis of the prevalence of pain in patients with bipolar disorder (12). This research contributes to the understanding of the association between pain and BD, emphasizing the need for integrated treatment approaches that address both psychiatric symptoms and pain management. Another meta-analysis by Stubbs B, published in the same year, discovered that the incidence of migraines is notably higher in patients with Bipolar Disorder (BD) than in the general population, particularly among those with Bipolar Disorder II (BD II) (29). Notably, the geographic location and diagnostic criteria used to define migraines influence the reported prevalence rates. Consequently, the study highlights the significance of employing standardized diagnostic criteria for migraines in BD research and the essential need for targeted therapeutic strategies and additional research in this domain.

Similarly, a dynamic partnership is observed between Abdin E, Chong SA and Subramaniam M. They co-authored a total of three articles, all of which are cross-sectional studies on Bipolar Disorder in Singapore.

Author co-citation analysis identifies scholars who are prominently cited within a specific domain (Figure 6B). Each node within the visualization corresponds to a scholar, with the lines between nodes indicating co-citation relationships between pairs of scholars. The node’s size directly correlates with the citation frequency of the represented author. Scholars characterized by a high volume of citations are thus recognized as pivotal figures within their field of study. As expounded in Table 3, Stubbs B emerges as the most frequently co-cited author (co-citation = 127), succeeded by Vancampfort D (co-citation = 96), and Kessler RC (co-citation = 86). Out of the 19,989 co-cited authors, six scholars received more than 100 co-citations.

### 3.5 Hotspots investigation

#### 3.5.1 Highly valuable papers

To evaluate the publications’ influence on osteoporosis research, we evaluated local citations. Table 4 lists the top 10 co-cited documents in the field of pain-related Bipolar Disorder. The top 10 papers primarily focus on the high comorbidity rates of pain in Bipolar Disorder, of which 4 articles pay particular attention to fibromyalgia.

**Table 4:** The top 10 documents with the most local citations in the field of pain-related bipolar disorder.

Rank	Title	LC	Journals	IF	PY	Author
1	The prevalence of pain in bipolar disorder: a systematic review and large-scale meta-analysis	27	Acta Psychiatrica Scandinavica	6.7	2015	Stubbs B
2	Pain conditions among veterans with schizophrenia or bipolar disorder	16	General Hospital Psychiatry	7.5	2013	Birgenheir DG
3	Comorbidity of Fibromyalgia and Psychiatric Disorders	14	J. Clin. Psychiatry	5.3	2006	Mayo Baltasar
4	Factors associated with pain interference in an epidemiologic sample of adults with bipolar I disorder	14	Journal of Affective Disorders	6.5	2009	Goldstein BI
5	Burden of general medical conditions among individuals with bipolar disorder	11	Bipolar Disord.	5.9	2004	Kilbourne AM
6	Pain perception in patients with bipolar disorder and schizophrenia	10	Acta Neuropsychiatr.	3.8	2007	Atik L
7	Prevalence, correlates, comorbidity and severity of bipolar disorder: Results from the Singapore Mental Health Study	9	J. Affect. Disord.	6.6	2013	Subramaniam M
8	Family study of fibromyalgia	9	Arthritis & Rheumatism	10.9	2004	Arnold LM
9	Prevalence of fibromyalgia and co-morbid bipolar disorder: A systematic review and meta-analysis	9	J. Affect. Disord.	6.6	2015	Kudlow PA
10	Fibromyalgia and bipolar disorder: a potential problem?	8	Bipolar Disord.	5.9	2019	Wilke WS

LC, local citation; PY, published year; IF: Impact Factor.

Moreover, references which garner widespread citation over time within a particular subject are identified as references with citation bursts. Serving as a valuable metric, these burst citations highlight references that have captured academic interest within a specified field during a certain timeframe. In this investigation, CiteSpace pinpointed the top 25 references bearing the most significant citation bursts, displayed in Figure 7B. Among these, the 2015 article authored by Stubbs B, which was mentioned above, held the highest rank (strength = 2.04) (12). Owen-Smith et al. examine the relationship between chronic pain, opioid prescriptions, and serious mental illnesses like major depressive disorder (MDD), bipolar disorder (BD), and schizophrenia (30). Individuals with MDD and BD have a higher likelihood of receiving chronic pain diagnoses and opioid prescriptions compared to the general population. Another reference with strong citation burst (strength = 1.88) discusses the complex associations between migraine and various psychiatric conditions such as major depression, bipolar disorder, anxiety disorders, and post-traumatic stress disorder (31). The study underscores the bidirectional relationship between these conditions and migraines, exploring potential shared genetic and environmental factors, as well as common neurobiological pathways.

Document Co-citation Analysis (DCA) holds significant importance as it provides a more comprehensive analysis through clusters based on various related domains (32).

There were 701 individual nodes and 1,822 links representing cited articles and co-citation relationships among the whole dataset, respectively. According to the narrative summary was drawn from the CiteSpace software, the network was divided into 181 clusters and the largest seven clusters were having the highest citation burst as well as citation count, indicating that these clusters were critical and active study efforts during the period of 2003–2023 on pain-related BP. Figure 7B represents some of the important clusters. The network’s overall mean silhouette value was 0.8646, which was a very high value representing homogeneity of clusters. As shown in Table 5, the range of silhouette values among the largest seven clusters were 0.948–0.993, which represents high homogeneity with each cluster. The table summarized the cluster size, silhouette value, mean year, citation count (cc), centrality (σ), citation burst, sigma value (Σ), and trend-setting cited references DOI number.

**Table 5:** The seven largest DCA clusters with top-5 most-cited references.

Cluster #	Cluster size	Silhouette value	Mean (year)	cc	Burst	σ	∑	DOI of cited references
0	64	0.949	2014	20	5.53	0.05	1.34	10.1111/acps.12325
				11	3.90	0.00	1.01	10.1002/wps.20252
				10	4.40	0.00	1.01	10.1002/wps.20420
				10	4.40	0.00	1.00	10.1002/wps.20309
				7	0.00	0.01	1.00	10.1016/j.schres.2014.10.017
1	44	0.977	2004	4	0.00	0.00	1.00	10.1038/nm1074
				3	0.00	0.00	1.00	10.1111/j.1528-1167.2005.463006.x
				2	0.00	0.01	1.00	10.1385/MN:32:2:173
				2	0.00	0.00	1.00	10.1016/j.eplepsyres.2005.02.002
				2	0.00	0.01	1.00	10.1016/j.bbr.2004.08.015
2	32	0.992	2015	5	0.00	0.00	1.00	10.1176/APPI.BOOKS.9780890425787
				3	0.00	0.03	1.00	10.1093/gerona/glv128
				3	0.00	0.00	1.00	10.1371/journal.pone.0063315
				3	0.00	0.00	1.00	10.1371/journal.pone.0114742
				3	0.00	0.00	1.00	10.1016/j.archger.2016.10.005
3	28	0.959	2013	5	0.00	0.02	1.00	10.1186/s12888-014-0350-4
				4	0.00	0.00	1.00	10.1186/1741-7015-11-263
				3	0.00	0.00	1.00	10.1097/PSY.0000000000000010
				2	0.00	0.00	1.00	10.1007/s11916-013-0356-5
				2	0.00	0.00	1.00	10.1016/j.jad.2010.07.004
4	25	0.967	2001	5	0.00	0.00	1.00	10.1097/00004714-200012000-00004
				4	0.00	0.00	1.00	10.1034/j.1399-5618.2000.20305.x
				3	0.00	0.01	1.00	10.1001/archpsyc.60.4.392
				3	0.00	0.01	1.00	10.4088/JCP.v60n0203
				3	0.00	0.01	1.00	10.1097/00004714-199908000-00010
5	20	0.993	1999	3	0.00	0.00	1.00	10.1093/hrp/9.5.209
				2	0.00	0.00	1.00	10.1016/S0920-1211(00)00196-0
				2	0.00	0.00	1.00	10.4088/JCP.v61n1106
				2	0.00	0.00	1.00	10.1097/00002508-200006001-00012
				2	0.00	0.00	1.00	10.1006/exnr.1999.7343
6	19	0.948	2016	6	0.00	0.01	1.00	10.1016/j.semarthrit.2016.08.012
				4	0.00	0.00	1.00	10.15585/mmwr.rr6501e1
				3	0.00	0.00	1.00	10.15585/mmwr.mm6626a4
				2	0.00	0.01	1.00	10.1016/j.mayocp.2016.04.029
				2	0.00	0.00	1.00	10.1007/s11926-016-0592-x

The data were derived from analysis (CiteSpace) of document co-citation network using dataset retrieved from Web of Science. cc-Citation count, σ-Centrality, Σ-Sigma. Cluster labelled by LLR (Log-Likelihood Ratio).

The main clusters were labeled as *exercise (#0), hyperandrogenemia (#1), sitting (#2), fibromyalgia (#3)*. Other significant clusters were *trigeminal neuralgia (#4; cluster size-25), mk-801 (#5; cluster size-20), and adverse selection (#6; cluster size-19).*

The most-cited references in the *exercise (#0; cluster size-64) were Stubbs, B_2015 (20), Vancampfort, D_2015 (11), Correll, CU_2017 (10), Vancampfort, D_2016 (10), Stubbs, B_2014 (7).* Cluster #0 explores the correlation between pain and bipolar disorder (BP). The most cited reference is still the one by Stubbs B mentioned earlier, which conducts a systematic review and meta-analysis of the prevalence of pain in patients with bipolar disorder. Moreover, it specifically pointed out the importance of recognizing specific types of pain, notably chronic pain and migraines, within the context of BP.

The second-largest cluster was *hyperandrogenemia (#1; cluster size-44) and the cited-references were Rogawski, MA_ 2004 (4), Amann, B_ 2005 (3), Bachmann, RF_ 2005 (2), Ahmad, S_ 2005 (2) and Arban, R_2005 (2)*. This cluster focuses primarily on the role and mechanisms of anticonvulsants in the treatment of bipolar disorder (BP). The literature in this cluster focuses on the treatment effects of anticonvulsants such as lamotrigine, valproate, and carbamazepine on the manic phases of bipolar disorder. Various studies, utilizing animal models or clinical data, have validated the effectiveness of these drugs in reducing manic symptoms and stabilizing mood fluctuations. Furthermore, through neurochemical analysis and neurophysiological studies, researchers have explored various mechanisms of action of anticonvulsants. This includes regulating neurotransmitter balance (such as enhancing GABA, inhibiting excessive glutamate release), ion channel modulation (especially sodium and calcium channels), and neuroprotective effects on neurons.

The third-largest cluster as *sitting (#2; cluster size-32) comprised 32 cited references, and the mean year was 2015.* The most-cited references of cluster #2 *were Association, AP_2022 (5), Garin, N_2016 (3), Freeman, EE_2013 (3), Koyanagi, A_2014 (3), and Zamora-Macorra, M_2017 (3)*. Further, the fourth-largest cluster in document co-citation analysis (DCA) is *fibromyalgia (#3; cluster size-28),* which involved 28 cited references and the mean year as 2013. The most-cited references of cluster #3 were *Nicholl, BI_2014 (5), Smith, DJ_2013 (4), Afari, N_2014 (3), Queiroz, LP_2013 (2) and Aguglia, A_2011 (2).* Cluster #3 discusses chronic multisite pain in bipolar disorder, particularly Fibromyalgia.

R package” Bibliometrix” identified the top 10 most co-cited references, which are exhibited in Table 6. The majority of these most co-cited references pertain to epidemiological discoveries about pain-related bipolar disorder, with a few studies focusing on specific types of pain in bipolar disorder, such as Migraine Headaches and Fibromyalgia.

**Table 6:** The top 10 most co-cited references in the field of pain-related Bipolar Disorder.

Rank	Title	TC	Journals	IF	PY	Author
1	The prevalence of pain in bipolar disorder: a systematic review and large-scale meta-analysis	118	Acta Psychiatrica Scandinavica	6.7	2015	Stubbs B
2	Depression and Pain Comorbidity: A Literature Review	98	Archives of Internal Medicine	8.0	2003	Bair MJ
3	Reliability and Validity of the Mini International Neuropsychiatric Interview for Children and Adolescents (MINI-KID)	89	J. Clin. Psychiatry	5.3	2010	Sheehan DV
4	A Rating Scale for Mania: Reliability, Validity and Sensitivity	76	British Journal of Psychiatry	10.5	1978	Young RC
5	Pain conditions among veterans with schizophrenia or bipolar disorder	70	Gen. Hosp. Psychiatry	7.0	2013	Birgenheir
6	The World Mental Health (WMH) Survey Initiative version of the World Health Organization (WHO) Composite International Diagnostic Interview (CIDI)	69	International Journal of Methods in Psychiatric Research	3.1	2004	Kessler RC
7	Comorbidity of Fibromyalgia and Psychiatric Disorders	63	The Journal of Clinical Psychiatry	5.3	2006	Arnold LM
8	Physical illness in patients with severe mental disorders. I. Prevalence, impact of medications and disparities in health care	54	World Psychiatry	73.3	2011	De Hert M
9	Factors associated with pain interference in an epidemiologic sample of adults with bipolar I disorder	51	Journal of Internal Medicine	6.6	2009	Goldstein BI
10	The Prevalence and Impact of Migraine Headache in Bipolar Disorder: Results From the Canadian Community Health Survey: CME	50	Headache: The Journal of Head and Face Pain	7.4	2006	McIntyre RS

TC, Total citation; PY, published year; IF: Impact Factor.

In summary, these highly valuable papers in this field identified through bibliometric analysis reveal researchers’ focus on particular types of pain associated with Bipolar Disorder, like fibromyalgia, migraines, and chronic pain. In fact, individuals with bipolar disorder are often diagnosed with these three types of pain. Exploring specific pain types in patients with bipolar disorder enables researchers to try creating more personalized treatment approaches for better managing their pain and emotional symptoms. However, this often requires collaboration across fields like psychiatry, neurology, and pain medicine. Further analysis on this will be conducted in the discussion section of our paper.

#### 3.5.2 Analysis of keywords

Keywords reflect the core or the main points the author wishes to express in an article. Therefore, keyword analysis in bibliometrics allows for exploration of hot topics and trends in the field. The keyword co-occurrence analysis facilitates the prompt identification of research focal points within a given area. Table 7 enumerates the 20 terms exhibiting the highest frequency within this field. The leading four keywords from the co-occurrence analysis include: bipolar disorder (133 occurrences), pain (93 occurrences), depression (87 occurrences), and prevalence (51 occurrences), all key terms associated with thematic research. It is noteworthy that “chronic pain” and “neuropathic pain” have appeared over 40 times, possibly indicating that the study of specific pain types in Bipolar Disorder is a primary research focus in this area.

**Table 7:** Top 20 keywords on research of pain-related Bipolar Disorder.

Rank	Keywords	Occurrences	Rank	Keywords	Occurrences
1	Bipolar disorder	133	11	Impact	32
2	Pain	93	12	Comorbidity	29
3	Depression	87	13	Psychiatric-disorders	27
4	Prevalence	51	14	Risk	23
5	Chronic pain	47	15	Major depressive disorder	22
6	Neuropathic pain	42	16	Schizophrenia	21
7	Double-blind	39	17	Risk-factors	21
8	Association	37	18	Major depression	20
9	Anxiety	33	19	Quality-of-life	20
10	Meta analysis	33	20	Health	19

Using a minimum co-occurrence threshold of 5 we included 193 keywords in a cluster analysis using VOSviewer. As depicted in Figure 5A the red cluster is very clear with keywords in this cluster primarily including “antiepileptic drugs,” “lamotrigine,” “anticonvulsants,” “gabapentin,” and “valproic acid.” Figure 5B displays high-frequency keywords in an overlay graph with the colors indicating the average publication year. Combining this with the red cluster in Figure 5A, it is evident that scholars have been actively investigating pain management strategies for Bipolar Disorder patients since 2012.

The keyword burst analysis by Citespace (Figure 7A) and the trend topic analysis by the R package “biblimetrix” (Figure 5C) allow us to understand the hot research areas of particular periods and the most recent trends in this research domain. In the keyword burst analysis (Figure 7A), the keyword “major depressive disorder” had the highest burst strength (strength = 2.4), with a burst period from 2022 to 2023. The term “childhood” exhibited the longest burst duration, spanning 3 years. Additionally, keywords emerging in the past 2 years, including psychological pain (strength = 1.36), perception (strength = 1.36), and adolescent (strength = 1.81), represent emerging fields.

## 4 Discussion

This investigation implemented sophisticated bibliometric analysis principles and advanced visualization techniques, utilizing tools such as VOSviewer, Citespace, the R package “bibliometrix,” and an online analysis platform. We executed comprehensive assessments of annual publication volume, geographic regions, and institutions, contributing authors and cited authors, academic journals and referenced journals, and pertinent keywords to unveil prevailing research focal points and tendencies in this domain.

### 4.1 General information

The annual publication volume of 9.1 from 2003 to 2009 indicates that research on pain-related Bipolar Disorder was in its initial stage at that time. Between 2010 and 2017, the publication volume in this field gradually increased, averaging 20.8 papers per year. In the period from 2018 to 2023, there was a marked increase in publication volume, averaging 42.2 papers per year. The swift increase in publications over the past 5 years highlights that pain research in bipolar disorder is in a period of explosive growth, gaining more and more interest from researchers.

The United States is the leading country in conducting research on pain in bipolar disorder, far ahead of other countries in both the number of publications (*n* = 226) and the cumulative citation frequency of these publications (citation = 10,190). Nevertheless, the United States shows a lack of active academic collaboration with other countries, potentially hindering its long-term progress in this area.

Regarding research institutions, the difference in cumulative publication volume among the top 10 is not significant, and they show a positive development trend in terms of annual publication output. There is close academic collaboration between South London and Maudsley University and King’s College London, as well as between the University of Pittsburgh and the University of Michigan.

Most research on pain-related Bipolar Disorder is published in the Journal of Affective Disorders, currently the most popular journal in this field. In terms of co-cited journals, the majority are high-impact Q1 journals. Clearly, these journals are high-quality international publications that provide support for research into pain related Bipolar Disorder.

In terms of authors, Stubbs B leads in publication volume and citations, establishing him as the most prominent author. Vancampfort D follows closely, with significant academic collaboration between the two authors. Similarly, there is a productive collaborative relationship between Abdin E, Chong SA, and Subramaniam M. Their publications have already been mentioned in the results section, and will not be elaborated on here.

### 4.2 Analysis of hotspots and frontiers in pain associated with bipolar disorder

Utilizing bibliometric tools to summarize the top co-cited documents and references with citation bursts, we have discerned highly valuable papers, predominantly concentrating on specific types of pain linked to Bipolar Disorder. Additionally, through analyzing the frequency of keyword occurrences, overlay displays, and burst detection results, we discovered that researching pain management strategies in bipolar disorder is a research hotspot and at the forefront of this field.

#### 4.2.1 Specific pain types in patients with bipolar disorder

The biopsychosocial model of pain depicts the physical manifestations of pain as outcomes of dynamic interplays between biological, psychological, and social elements (33). How pain is categorized influences prognosis, diagnostic assessments, and treatment across different stages. Therefore, focusing on the types of pain associated with bipolar disorder helps in studying the comorbid pathologies of pain and bipolar disorder, aiding in the development of more effective pain management plans for BD patients, thereby improving their treatment and quality of life. Based on the most common keywords, research into specific types of pain is largely concentrated on “chronic pain,” “neuropathic pain,” “fibromyalgia,” and “migraine.”

##### 4.2.1.1 Chronic pain

The International Association for the Study of Pain (IASP) characterizes chronic pain as an unpleasant sensation and emotional experience linked to, or resembling, actual or potential tissue damage. Chronic pain is prevalent among individuals with mood disorders (34), including major depressive disorder (MDD), anxiety disorders, and bipolar disorder. A large-scale (*N* = 106,214) meta-analysis suggests that 23.7% of BD patients may have chronic pain, and this seems to be related to sleep disorders and delayed diagnosis of BD (30). In a cross-sectional study conducted by Nicholl et al., it was found that chronic pain has a strong association with BD, even after adjusting for potential confounding factors such as age, gender, race, socioeconomic status, employment status, BMI, smoking, and alcohol consumption (35).

Individuals with Bipolar Disorder and concurrent chronic pain encounter challenges, as comorbid pain significantly impacts their quality of life. Patients suffering from bipolar disorder and chronic pain typically exhibit poor responses to treatment and face an elevated risk of suicide (7, 36). A study assessing the relationship between pain and bipolar disorder suggests incorporating pain assessments as a part of routine care for bipolar patients (7). Adequate treatment of these patients requires an interdisciplinary approach. Besides pain specialists, psychotherapists are also a crucial part of the treatment team.

##### 4.2.1.2 Migraine

Migraine, an episodic neurological disorder, involves severe headaches usually concentrated on one side of the head (36). Symptoms commonly include nausea, vomiting, dizziness, visual problems, and sensitivity to light and sound (37). Approximately 16% of the general population is affected by migraines, while recent evidence indicates that around 25% of individuals with bipolar disorder suffer from migraines (29). The most frequent type of pain in bipolar disorder patients with migraines is throbbing pain (63.7%) (38). Compared to those with only bipolar disorder, the coexistence of bipolar disorder and migraines is often associated with higher rates of depressive episodes and increased suicide risk (38).

Currently, there is limited knowledge regarding the potential pathological mechanisms connecting bipolar disorder with migraines. The comorbidity of migraines and bipolar disorder might be due to dysfunctions in neurotransmitters within the shared serotonergic, dopaminergic, and glutamatergic pathways of the two conditions. Additionally, some studies indicate the presence of common genetic abnormalities between migraines and bipolar disorder (39).

##### 4.2.1.3 Fibromyalgia

Fibromyalgia (FM), a form of Nociplastic pain, is a central pain syndrome marked by chronic widespread pain along with fatigue, sleep disorders, and cognitive dysfunction (40). Fibromyalgia (FM) is often confused with the pain perception in bipolar disorder (BD). The association between the two and their shared pathophysiology is a current research focus. Current research indicates that these two disorders share epidemiological characteristics and might have common biological pathways (41).

First, both FM and BD exhibit symptoms of chronic widespread pain, fatigue, sleep disturbances, and cognitive impairments. This clinical overlap suggests a potential connection at the neurobiological level. Brain imaging research supports the structural and functional similarities between FM and BD. Specifically, MRI in FM patients reveals reduced gray matter density in brain areas linked to pain transmission and processing (42), while functional MRI studies show enhanced activation in the pain matrix regions of their brains (43). In contrast, imaging studies in BD patients indicate certain structural and functional abnormalities in the prefrontal cortex, and an increased volume of the amygdala, which is associated with high activity levels during mood elevation phases (44). Furthermore, studies have identified that the impaired stress responses linked to FM and BD, and possible hypothalamus-pituitary–adrenal (HPA) axis dysfunctions, are common pathophysiological characteristics shared by both disorders (45, 46).

The high comorbidity rate of FM in BD patients gives research into the treatment of both conditions significant clinical importance. Overactive neurotransmitters are thought to be a cause of Fibromyalgia, making it difficult to treat these neurotransmitters when one is also taking medication for bipolar disorder. The reason lies in the use of SSRIs (Selective Serotonin Reuptake Inhibitors) to suppress neurotransmitter activity, known for inducing mania. Comprehensive assessment and treatment of patients with comorbid FM and BD, particularly in terms of medication selection and psychosocial interventions, may lead to the discovery of new drug targets, aiding in the development of more effective treatment strategies.

#### 4.2.2 Pain management of bipolar disorder

Keywords like “antidepressant,” “marijuana use,” “social support” in strong citation burst analysis, along with “lamotrigine,” “antiepileptic drug,” “opioids” in trend topic analysis, suggest an increasing research interest in pain management in patients with bipolar disorder. Managing pain in bipolar disorder patients may require a multidisciplinary approach, including both pharmacological and non-pharmacological treatments.

##### 4.2.2.1 Pharmacological treatments

In the treatment of bipolar disorder patients with comorbid pain, mood stabilizers often serve as the main therapeutic approach, occasionally also providing relief from headaches. The three types of mood stabilizers include anticonvulsant drugs, lithium, and atypical agents.

Lamotrigine, an anticonvulsant, is frequently utilized as an antiepileptic in bipolar disorder (47). Its versatility extends to treating certain types of neuropathic pain, although it is ineffective for headaches. Sodium valproate is effective for mania, hypomania, bipolar depression, and migraines but can lead to birth defects at daily doses above 500 mg (48). In this context, Lamotrigine, by comparison, may be a preferable antiepileptic during pregnancy (49). Divalproex sodium is believed to function by increasing levels of gamma-aminobutyric acid (GABA) in the brain, used for treating manic phases of bipolar disorder, and is also one of the most common medications for migraine prevention (50). Carbamazepine, an anticonvulsant related to tricyclic antidepressants, is one of the few mood stabilizers recognized in American Psychiatric Association guidelines as effective for chronic pain. While it can relieve neuropathic pain as a mood stabilizer but does not prevent migraines (51, 52). Gabapentin, in high doses, can be effective for patients with mild bipolar disorder (53). Notably, it works for some pain syndromes, particularly in alleviating some forms of neuropathic pain, but is not effective for migraines (54).

Lithium is frequently used in the treatment of multiple sclerosis and dementia, and it is a first-line medication for manic episodes. Moreover, it has also proven effective in preventing relapses of depression and reducing suicide rates (55). Studies have discovered that lithium can relieve cluster headaches (56). However, a frequent drug interaction concern for bipolar disorder patients is lithium toxicity. When appropriately monitored, low doses of lithium usually do not lead to thyroid dysfunction or kidney stimulation (57).

##### 4.2.2.2 Non-pharmacological treatments

Bipolar Disorder patients have various non-medication pain management options, including Mindfulness-Based Cognitive Therapy (MBCT), Cognitive Behavioral Therapy (CBT), and complementary alternative methods such as acupuncture and massage.

MBCT addresses pain by leveraging awareness of the present moment. Studies show that MBCT interventions demonstrate clinical efficacy in a wide range of chronic pain conditions beyond fibromyalgia and back pain (58). Mindfulness combined with CBT provides superior relief from migraine symptoms and enhances life quality by addressing sensory, cognitive, and emotional elements. The mechanisms through which MBCT alleviates pain are multifaceted. Neuroimaging studies suggest that mindfulness might involve top-down modulation from the prefrontal cortex and anterior cingulate cortex to suppress maladaptive pain signals (59). Additionally, evidence indicates that mindfulness affects pain-regulating neurotransmitters (60). Genomic and proteomic studies suggest that mindfulness can reduce the expression of inflammatory genes and proteins, mitigating peripheral and central sensitization (61).

Cognitive Behavioral Therapy (CBT) is a psychological therapy that aims to enhance mood and alleviate symptoms by modifying patients’ thought processes and behaviors. It has been proven effective for bipolar disorder, fibromyalgia, migraines, and neuropathic pain (62). Similar to MBCT, Cognitive Behavioral Therapy also modulates the activity and connectivity of brain regions involved in pain processing (including the prefrontal cortex, anterior cingulate cortex, insula, and amygdala). Moreover, CBT regulates pain using a “gate control” mechanism, engaging the descending modulation pathway that releases neurotransmitters like opioids, serotonin, and norepinephrine, thereby inhibiting harmful signal transmission in the spinal cord (63). Cognitive-behavioral interventions have demonstrated high safety and tolerability.

Acupuncture regulates pain signals by stimulating traditional acupoints along the meridians. Studies show it achieves pain relief across diverse chronic pain conditions, with clinical trials revealing its efficacy in managing cancer-related pain, menstrual cramps, migraines, tension headaches, abdominal pain, and chemotherapy-induced peripheral neuropathy (64, 65). Additionally, acupuncture produces beneficial synergistic effects through various regulatory mechanisms and in combination with other treatment methods (65). However, limitations of acupuncture therapy, such as varying clinical effectiveness for different pain disorders and individuals, risks of infection from single-use needles, and issues like pneumothorax, are worth considering.

In summary, there is currently no consensus on guidelines for pain management in patients with bipolar disorder. The choice of pain treatment and medication specifically depends on whether it involves symptoms (such as fibromyalgia) or conditions (like migraines). Although pain treatment based on comorbid mechanisms is ideal, identifying underlying mechanisms in clinical practice can be challenging or impossible, hence treatments are often based on symptoms. For numerous patients, treatment objectives should be tailored to individual circumstances. Enhancing the quality of life might be more practical than significant pain reduction.

## 5 Conclusion

Our bibliometric analysis provides an overview of the current state and emerging trends in pain-related BD research from 2003 to 2023. The findings of this study could assist in broadening treatment plans for BD patients with pain and establish a groundwork for additional research in this domain. The importance of pain in BD is increasingly recognized. However, this field still requires comprehensive exploration, and a deeper investigation into the pathophysiological mechanisms of different pain types in BD patients could aid in developing pain management strategies for them. We look forward to more high-quality future research to improve treatment strategies for BD patients with pain and enhance their quality of life.

## Data availability statement

The datasets presented in this study can be found in online repositories. The names of the repository/repositories and accession number(s) can be found in the article/Supplementary material.

## Author contributions

HZ: Writing – original draft. MZ: Software, Writing – review & editing. JJ: Conceptualization, Writing – original draft. ZL: Writing – original draft. YW: Writing – review & editing.

## Footnotes

^1^https://www.bibliometrix.org
